# CD3+ T-cell count prediction for anti-thymocyte globulin treatment monitorization in kidney transplant recipients: a machine learning model

**DOI:** 10.3389/fmed.2026.1869846

**Published:** 2026-06-18

**Authors:** Ahmet B. Ak, Hayri K. Goren, Nuri B. Hasbal, Nur I. Genc, Sidar Copur, Lasin Ozbek, Burak Kocak, Adrian Covic, Mehmet Kanbay

**Affiliations:** 1 Department of Internal Medicine, Koc University School of Medicine, Istanbul, Türkiye; 2 Division of Nephrology, Department of Internal Medicine, Koc University School of Medicine, Istanbul, Türkiye; 3 Koc University, School of Medicine, Istanbul, Türkiye; 4 Department of Urology, Koc University School of Medicine, Istanbul, Türkiye; 5 Munci Kalayoglu Organ Transplant Center, Koc University Hospital, Istanbul, Türkiye; 6 Nephrology Clinic, Dialysis and Renal Transplant Center—'C.I. Parhon' University Hospital and 'Grigore T. Popa' University of Medicine, Iasi, Romania

**Keywords:** antithymocyte globulin, ATG, CD3+ T-cell, immunosuppression therapy, kidney transplant, machine learning, T-lymphocytes

## Abstract

**Background and aim:**

Antithymocyte globulin (ATG) therapy is conventionally monitored by measuring peripheral blood CD3+ T-cell counts. However, CD3+ T-cell quantification requires flow cytometry and may not be routinely available in centers. This study aimed to identify clinical and laboratory predictors of CD3+ T-cell depletion and to develop a machine learning model for predicting attainment of predefined therapeutic CD3+ T-cell thresholds following ATG induction therapy in kidney transplant recipients.

**Materials and methods:**

Adult transplant patients who underwent kidney transplantation were retrospectively evaluated. ATG doses were administered as induction therapy, and the subsequent peripheral blood CD3+ T-cell counts were obtained. Demographic, anthropometric features, and laboratory results were recorded. Statistical and machine learning methods were applied to identify parameters predictive of CD3+ T-cell counts. Prediction models were developed and compared to the logistic regression model to estimate the likelihood of reaching certain CD3+ T-cell count cut-offs after transplantation to evaluate and monitor ATG effect.

**Results:**

In the analysis of 397 transplant patients of 99.2% grafts from living donors, 57.2% of patients achieved the predefined day-1 CD3+ T-cell < 50 cell/μl threshold, while 57.5% of patients reached the predefined day-2 CD3+ T-cell < 30 cell/μl threshold. The machine learning model’s performance in predicting target threshold attainment resulted in ROC-AUC values of 0.75 and 0.80 for Day 1 (test and validation sets), whereas Day 2 predictions yielded ROC-AUC scores of 0.70 and 0.66, respectively. The predictive performance of the machine learning model was superior to logistic regression prediction, and decision curve analysis showed that the model provided clinically meaningful net benefit for decision-making for Day 1 and Day 2 predictions.

**Conclusion:**

The clinical response to ATG treatment for induction immunosuppression in kidney transplant patients may be adequately predicted using low-cost and accessible laboratory tests alongside patient-specific characteristics without the need for CD3+ T-cell quantification, through machine learning prediction models. This study provides an initial framework for further development of more accurate machine learning models with higher prediction power for clinical use in ATG effect prediction.

## Highlights

- *What was known*: ATG is one of the commonly used induction treatment agents in kidney transplantation. Monitoring optimal ATG dosing is important for preventing undertreatment, which could result in acute rejection, while preventing overtreatment, which could lead to infection, malignancy, and mortality. The mainstay method to monitor ATG treatment is CD3+ T-cell counting with the use of a flow cytometer.
- *This study adds*: A machine learning model for predicting CD3+ T-cell counts is a cheaper and robust alternative for guiding and tailoring ATG treatment, especially in low-resource settings.
- *Potential impact*: Machine learning tools to predict treatment effect would be helpful for monitoring ATG treatment without the need for CD3+ T-cell measurements. Optimal management of induction immunosuppression would prevent mortality, graft loss, infection, malignancy risk, and the high cost of ATG treatment and associated complications comes with over or undertreatment of ATG.

## Introduction

Kidney transplantation is currently the mainstay of effective kidney replacement therapy for patients with end-stage kidney disease (1). Upon transplantation, induction immunosuppressive treatment is necessary to prevent acute rejection of the transplanted graft, especially in immunologically high-risk transplantations (2). Antithymocyte globulin (ATG) is an efficacious option for induction therapy in kidney transplantation (1) and works through lymphocyte depletion that orchestrates the immunologic destruction of the foreign-considered graft kidney by causing complement-mediated lysis or direct activated apoptosis on T-lymphocytes (3).

In kidney transplantation, the risk of acute rejection is significantly elevated when adequate immunosuppressive induction with ATG is not achieved. Conversely, supratherapeutic administration of ATG may precipitate adverse outcomes, including increased risk for patient morbidity and mortality (4, 5) by elevating the risks of infection (6) and malignancy (7) associated with excessive immunosuppression. For this reason, the monitoring of appropriate ATG administration is of paramount importance (8).

ATG treatment monitoring and follow-up are conducted based on serum CD3+ T-cell measurement (9). CD3+ T-cells are deemed to be the main mediators for acute allograft rejection. A recent study demonstrated that acute rejection rates to be higher with patients having higher CD3+ T-cell counts, hence lowering CD3+ T-cells to a certain level is accepted as the current standard for adequate induction immunosuppression (10, 11). Other determinants employed in research, including absolute lymphocyte counts, show certain prediction strength but are not ideal for monitoring ATG treatment in a generalizable and precise manner (11, 12). Consequently, given that the determination of CD3+ T-cell levels is costly and necessitates specific infrastructure, the identification of alternative methods capable of safely monitoring the administration of ATG at the appropriate dosage and duration is a prominent area of investigation.

Machine learning (ML) algorithms are becoming increasingly prevalent to obtain prediction models and are gaining prominence over conventional statistical methods owing to their capacity to facilitate processing and their superior modeling capabilities when dealing with large-scale and high-dimensional data. ML tools are gaining more ground within the field of transplantation, paralleling their application in personalized medicine (13).

In this study, we aim to monitor ATG therapy by employing a machine learning model predicting whether given ATG treatment is optimal to reach predefined CD3+ T-cell count cut-offs by evaluating patient characteristics and laboratory results other than CD3+ T-cell count.

## Materials and methods

### Study design and patient population

**Figure 1:**
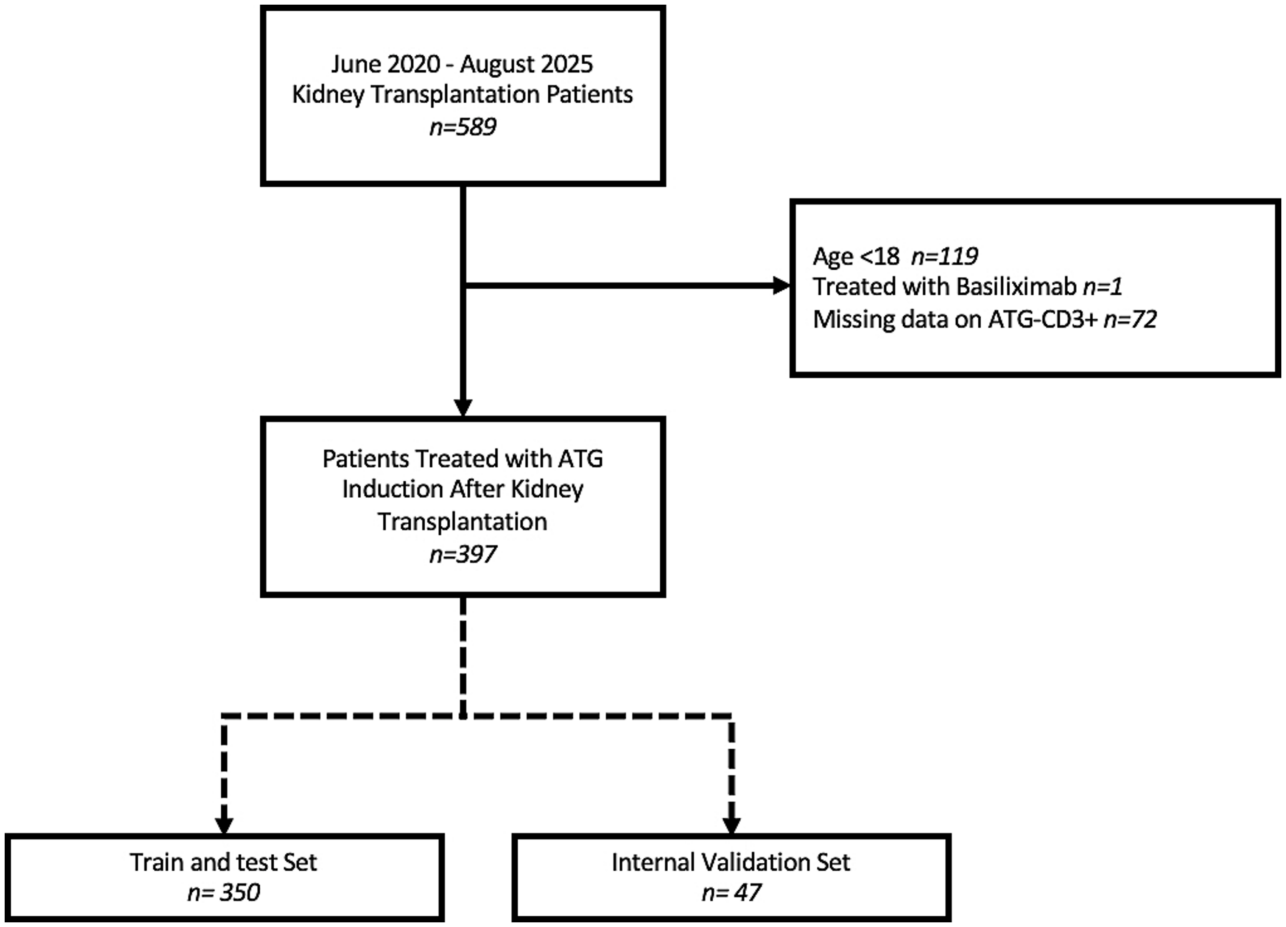
Process of patient selection and inclusion.

In this retrospective cohort study, we reviewed patients who underwent kidney transplantation in Koc University Hospital between 01.06.2020 and 31.08.2025. A total of 589 patients were investigated. Exclusion criteria for the study included (a) age <18 years, (b) induction therapy with non-ATG regimens, and (c) missing data regarding ATG treatment or CD3+ T-cell counts. The study was carried out with ethical approval obtained from Koc University institutional board of ethics (Koc University Biomedical Research Ethics Committee Institutional Review Board 2025.392. IRB1.063). The patient enrollment and selection process is depicted in Figure 1.

Patient demographic (sex, age) and anthropometric characteristics [height, weight, body mass index (BMI), body surface area (BSA)]; antithymocyte globulin (ATG) doses administered within the first 3 days; preoperative and postoperative day-1 levels of alanine aminotransferase (ALT), aspartate aminotransferase (AST), alkaline phosphatase (ALP), and gamma-glutamyl transferase (GGT); preoperative serum creatinine; serum tacrolimus levels; and leukocyte, lymphocyte, and platelet counts obtained from complete blood counts performed one day prior to surgery and on the day of surgery. We evaluated which variables derived from patient-specific data obtained during the routine transplantation process could provide accurate predictions of CD3+ T-cell counts as a measure of ATG response, as well as the predictive strength of these results.

The primary endpoint was defined as the achievement of the target CD3+ threshold on postoperative days 1 and 2, which was established as <50 cells/μl for the first postoperative day and <30 cells/μl for the second day, determined based on existing literature, which utilizes target ranges of <20–100 cells/μl for dose adjustment in order to prevent acute rejection following ATG therapy (14–16). In our center, we routinely monitor all kidney recipients with CD3+ T-cell count upon ATG administration. The <50 and <30 cells/μl cut-offs were selected in accordance with the established transplant protocol in our institution, developed based on current evidence and clinical experience. Flow cytometric analyses were performed using a BD FACSLyric™ flow cytometer and FACSuite™ software.

ATG therapy, initiated on the day of surgery, was administered as a once-daily infusion for three consecutive days. However, treatment was suspended before the scheduled 3 days in the event of limiting adverse effects, such as leukopenia lower beyond the target threshold or severe thrombocytopenia. All the patients were treated with the same triple immunosuppressive regimen for prevention of graft rejection and triple antibacterial, antiviral, and antifungal prophylaxis.

### Statistical analyses and machine learning model

Statistical analyses were performed using IBM SPSS version 28.0.0.0. Categorical data were presented as percentages, while continuous data were presented as mean (standard deviation).

Machine learning models were developed using supervised learning methods. Feature engineering was employed to reduce data noise; variables with high rates of missing data (e.g., gamma-glutamyl transferase) and parameters such as height and weight, which are inherently represented within the body surface area derivative, were excluded from the model. Furthermore, to enhance precision, derivative ratios were generated, such as the platelet ratio (Day −1 count / Day 0 count) and the leukocyte ratio (Day −1 count / Day 0 count). Patient outcomes were categorized using a binary classification based on whether CD3+ T-cell count targets were achieved following the initial ATG administration (Day 1: <50 cells/μl; Day 2: <30 cells/μl).

Patients were randomly allocated into training and testing sets at an 8:2 ratio (280 patients on the training set, 70 patients on the test set). Following this split, data preprocessing and iterative imputation for missing values were performed, excluding the target variables (ATG dosage and CD3+ T-cell counts). The iterative imputation model was fitted and transformed on the training set to prevent data leakage, while the test set was processed using a transformation-only approach. This provided that missing values in the test set were imputed based solely on the parameters learned from the training data. Before imputation, the number of missing values for each variable was 0.75% for ALT, 0.50% for AST, 43.82% for GGT, 1.51% for ALP, and 0.25% for trough tacrolimus concentration. All remaining variables contained complete data and required no imputation. The random state was set to “42” to ensure reproducibility. We chose automated ensemble learning rather than single model regression. For this purpose, modeling was conducted using the AutoGluon Tabular Predictor library. This method automatically tested multiple tree-based models using bootstrap aggregating (bagging) and stacking-based ensemble techniques to select the models yielding optimal results. Cross-validation was performed via AutoGluon’s automated stratification methods. Consequently, models possessing the hyperparameters necessary for ideal prediction were integrated to combine predictor features. To evaluate machine learning performance on datasets, specificity, sensitivity, positive predictive value (PPV), negative predictive value (NPV), accuracy, receiver operating characteristic (ROC) curves, area under the curve (AUC), and Youden’s index were utilized. The variables contributing most significantly to the model’s predictions were ranked according to their importance scores using AutoGluon’s feature importance tool.

The model was trained utilizing hyper parameters optimized to yield the highest predictive accuracy. The area under the receiver operating characteristic curve (ROC-AUC) served as the primary metric to demonstrate the predictive performance of the trained model on the validation set. In addition to standard performance parameters, SHAP analysis, calibration curves, and decision curve analysis (DCA) were conducted on both test and validation sets to evaluate the model’s clinical utility and explainability. Bootstrap resampling was performed to generate confidence intervals, thereby assessing the statistical uncertainty and stability of the model.

To compare the predictive power of the machine learning model against conventional statistical methods, standard logistic regression analyses were performed for Day 1 and Day 2 outcomes. Python 3.11.12 (Python Software Foundation) and *Scikit-learn* library were utilized for data preprocessing, editing, and performance metrics, while Google Colaboratory (Google, 2025) was employed for the execution of machine learning algorithms.

## Results

### Population demographics and baseline values

397 patients were included in the study (Figure 1). Among those, 3 patients underwent deceased donor kidney transplantation, while the remaining 394 received grafts from living donors. The dataset comprising the first 350 patients, ordered chronologically according to their transplant surgery date, was utilized for the train and test evaluations of the prediction model. The remaining 47 patients, excluded completely from the training set, were used as an internal validation dataset to evaluate the model. All patients were evaluated regarding ATG doses administered for induction therapy; ATG regimens administered as rescue therapy for rejection were excluded from the analysis. Demographic data, anthropometric measurements, and laboratory results of the patients are presented in Table 1.

**Table 1:** Demographics, clinical and laboratory parameters of included patients.

Clinical and laboratory parameters	Patients (*n = 397*)
Gender, *n* (%)	
Male	260 (65.5)
Female	137 (34.5)
Age, mean (SD)	43.77 (13.56)
BMI (kg/m^2^), mean (SD)	25.48 (4.99)
Graft origin, *n* (%)	
Cadaveric	3 (0.8)
Living	394 (99.2)
ATG dose (mg), mean (SD)	
Day 1	221.98 (53.03)
Day 2	238.39 (62.80)
Day 3	229.59 (77.70)
CD3+ T-cell count (cell/μl), n (%)	
Day 1 < 50 target achieved	227 (57.2)
Day 2 < 30 target achieved	204 (57.5)
Blood count parameters day −1 (x10^3^/μl), mean (SD)	
Leukocyte	7.20 (2.49)
Lymphocyte	1.73 (0.70)
Thrombocyte	204.27 (130.09)
Blood count parameters day 0 (x10^3^/μl), mean (SD)	
Leukocyte	9.39 (5.05)
Lymphocyte	0.12 (0.11)
Thrombocyte	0.12 (0.11)
Tacrolimus level (ng/mL), mean (SD)	8.25 (4.58)
Serum transaminase level (U/L), mean (SD)	
ALT	26.87 (40.08)
AST	32.51 (32.99)
ALP	75.18 (66.64)
GGT	35.61 (34.54)

ALP, alkaline phosphatase; ALT, alanine aminotransferase; AST, aspartate aminotransferase; ATG, antithymocyte globulin; BMI, body mass index; CD, cluster of differentiation; GGT, gamma-glutamyl transferase; SD, standard deviation; n, count.

The population observed to be 65.5% male, with a mean age of 43.7 years and a mean BMI of 25.48 kg/m^2^. The mean administered ATG doses were 221.98 mg on Day 1, 238.39 mg on Day 2, and 229.59 mg on Day 3. Decreasing lymphocyte and platelet levels were observed with ATG treatment as expected. The mean tacrolimus level was 8.48 ng/mL, which was considered consistent within the target range of 8–12 ng/mL for the early post-transplant period. Mean transaminase levels were observed to be within normal reference ranges.

### CD3+ T-cell count prediction analyses

ROC-AUC scores were prioritized to evaluate the model’s true predictive performance regarding the achievement of CD3+ T-cell counts <50/μl on Day 1. The model yielded an ROC-AUC score of 0.75 in the test dataset and 0.80 in the validation dataset. For the prediction of CD3+ T-cell counts <30/μl on Day 2, ROC-AUC scores were 0.71 in the test dataset and 0.65 in the validation dataset. In comparison, predictions utilizing logistic regression yielded ROC-AUC scores of 0.71 (test) and 0.66 (validation) for Day 1. For Day 2 predictions, logistic regression resulted in ROC-AUC scores of 0.61 in the test set and 0.48 in the validation set. In addition to ROC-AUC scores, sensitivity, specificity, PPV, NPV, accuracy, and Youden index values are presented in Supplementary Table S1.

To assess model stability, 95% confidence intervals (CI) for ROC-AUC were derived using a 1,000-iteration bootstrap resampling method. Upon examination of the post-resampling results, the machine learning model demonstrated superior predictive power compared to logistic regression. The mean ROC-AUC values for the machine learning model were 0.75 (test set) and 0.80 (validation set) for Day 1, and 0.70 (test set) and 0.66 (validation set) for Day 2. In comparison, the ROC-AUC values for the logistic regression model were observed to be inferior to those of the machine learning model for both days. For Day 1, the logistic regression model yielded ROC-AUC values of 0.71 in the test set and 0.67 in the validation set. For Day 2, the ROC-AUC score was 0.61 in the test set, whereas it resulted in 0.41 in the validation set (Supplementary Table S2).

**Figure 2:**
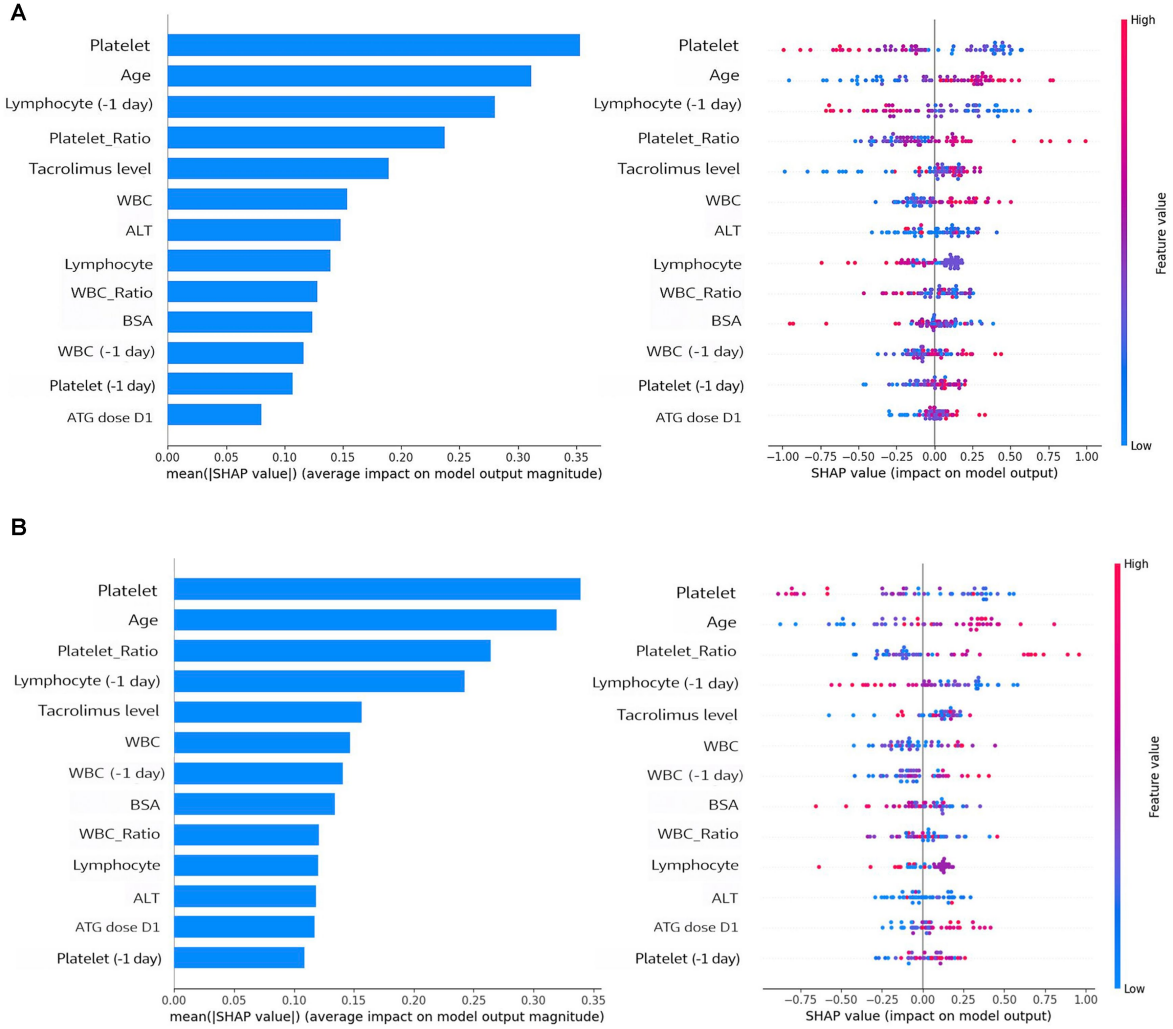
**(A)** SHAP analysis and beeswarm plot of AutoGluon model for day 1 on test set and **(B)** on validation set. The left panel shows feature importance ranking based on mean absolute SHAP values, whereas the right panel represents the SHAP beeswarm summary plot demonstrating the direction and magnitude of each feature’s contribution to model predictions.

**Figure 3:**
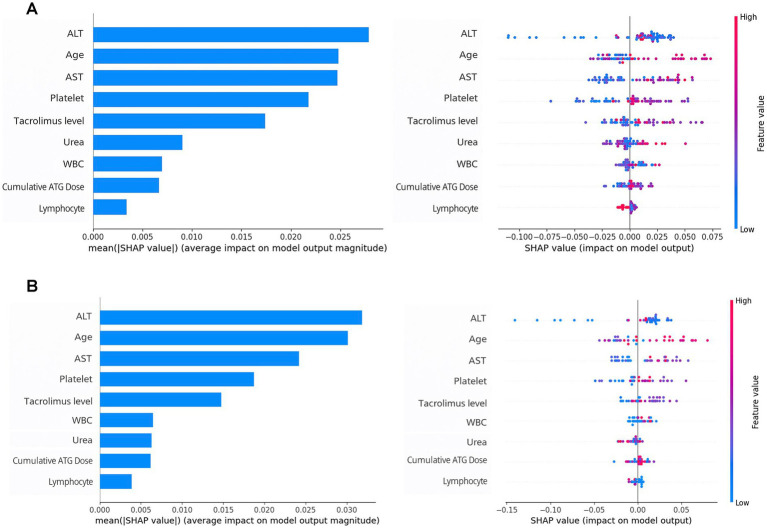
**(A)** SHAP analysis and beeswarm plot of AutoGluon model for day 2 on test set and **(B)** on validation set. The left panel shows feature importance ranking based on mean absolute SHAP values, whereas the right panel represents the SHAP beeswarm summary plot demonstrating the direction and magnitude of each feature’s contribution to model predictions.

To evaluate the clinical net benefit of the ensemble model prediction decisions, reliability of predicted results, biological and clinical interpretability of the model; decision curve analysis (DCA), calibration curve, and SHAP (SHapley Additive exPlanations) analysis were utilized on the highest-performing AutoGluon model (Figures 2 and 3, Supplementary Figures S1–S4). Through the resulting ranking of feature importance, the specific parameters influencing clinical decision-making for the patient cohort are visualized on SHAP analysis.

## Discussion

This study demonstrates an effective model to predict whether a patient treated with ATG might reach CD3+ T-cell <50/μl on Day 1, with ROC-AUC scores for Day 1 being 0.75 (95% CI 0.63–0.86) and 0.80 (95% CI 0.65–0.93) in the test and validation sets, respectively. Prediction of reaching CD3+ T-cell <30/μl on Day 2 was slightly less powerful but showed promising prediction performance for day 2, with ROC-AUC scores being 0.70 (95% CI 0.57–0.83) and 0.66 (95% CI 0.41–0.87) in the test and validation sets, respectively.

For Day 1, the analysis indicated that the ML model’s predictive power was primarily derived from the platelet count, patient age, lymphocyte count on the day prior to ATG administration, the ratio of platelet counts between the preceding day and the treatment day, and tacrolimus levels, respectively. Low platelet count on day 0, high age, high platelet ratio (Day −1 count / Day 0 count), and low lymphocyte count on day −1 were associated with a higher chance of reaching the ATG treatment goal. This result is correlated with the expected leucopenia and thrombocytopenia effect of ATG, even in therapeutic doses (17).

Transaminase levels and kidney function, along with the patient’s weight, BMI, and BSA, were included as parameters in the model, considering their potential determining and surrogate roles in reflecting the patient’s metabolism, hence the changes in ATG level. On day 1, where the model provided the strongest prediction with the abundance of data, the main determinants were complete blood count, age, and tacrolimus level, while transaminase levels and kidney functions were not on the list of primary sources of predictors. There is insufficient information regarding whether ATG, a polyclonal antibody, is metabolized by the kidneys and liver at all; the fact that ATG breakdown occurs in the reticuloendothelial system may explain this finding.

For day 2, the model prediction was mainly powered by ALT, age, AST, platelet count, and tacrolimus measurement. Following the initial dose of ATG, a marked reduction in lymphocyte and platelet counts was observed compared to the preceding day. This is thought to be the source of the low prediction performance of the model on Day 2. Minuscule measurable differences in blood count parameters among patients potentially narrow the detection capacity of associations. Hence, the model uses other parameters, that is unexpected to be related to ATG, such as AST, ALT levels, to find associations with CD3+ T-cell count.

Machine learning methods were employed to evaluate the relationship between the available data and CD3+ T-cell levels in a multi-layered manner. This approach was intended to capture non-linear relationships that might be overlooked by essential classification algorithms such as logistic regression (LR) analysis, thereby achieving more accurate predictions. The use of AI-based machine learning models in organ transplant processes is becoming increasingly widespread. Machine learning and AI models are effectively used to predict patients’ potential hospitalization needs due to post-transplant complications (18), to monitor complications (19), to assess graft loss risks (20), and to estimate the likelihood of recurrence of the primary disease (21). A recent study applied machine learning approaches to characterize post-transplant immune reconstitution and establish reference values for lymphocyte subpopulations in kidney transplant recipients under both stable and infectious conditions over the long term (22). In addition, the authors confirmed previous findings that ATG exposure is the strongest determinant of long term CD4+ T-cell recovery, suggesting that optimization of ATG dosing may influence not only early postoperative immune dynamics but also long-term clinical outcomes (15, 22).

Our study has several limitations. First, as this was a single-center study, the dataset was derived from a relatively small patient cohort. Data from a higher number of patient population with more parameters to be tested for ATG effect would result in more accurate and powerful predictions for ATG treatment. Secondly, since the findings rely on a single-center retrospective analysis, external validation utilizing data from external cohorts could not be performed to verify the predictive performance of the model. Thirdly, as the nature of a retrospective analysis, the etiology of primary kidney disease was unknown in a significant proportion of transplant recipients; consequently, the potential impact of concomitant immunological, rheumatological, or hematological disorders on biochemical and blood count parameters could not be evaluated. The predictive power of the model could potentially be enhanced by incorporating the presence of primary pathologies capable of altering baseline blood count parameters, evaluating the efficacy of ATG relative to baseline CD3+ levels, and including patient comorbidities and routine medication usage, thereby yielding more accurate predictions. Finally, the majority of the patient population was male. Due to the risk of introducing heterogeneity, biological sex was not included as a predictive factor. However, given the potential for metabolic differences between males and females, it is suggested that more accurate results could be achieved using datasets from a more balanced patient population where this determinant is incorporated. Additionally, as the vast majority of cases involved living-donor transplantation, the current model and dataset provide insufficient insight into the distinct metabolic and immunological factors that might influence ATG response predictions in deceased-donor kidney recipients. Since the patient population was limited, with ATG treatment used only for the induction regimen of kidney transplantation; the success of the model might not be valid for the rejection treatment of ATG. Moreover, randomized controlled trials with long-term follow-up are required to draw conclusions regarding graft survival and mortality, given the absence of such follow-up data in this retrospective analysis.

In this study, we demonstrated a clinically beneficial, effective machine learning tool to predict therapeutic ATG dosing for guidance without measuring CD3+ T-cells in a cohort consisting predominantly of living donor kidney transplant patients. This approach seeks to utilize relatively less expensive, accessible, and patient-oriented data and measurement results, thereby providing personalized patient monitoring with a decrease in the need for other specialized assays in optimizing ATG treatment.

## Data Availability

The raw data supporting the conclusions of this article will be made available by the authors, without undue reservation.

## Supplementary material

SUPPLEMENTARY FIGURE S1 Calibration curve of AutoGluon model for day 1 on test set **(A)** and validation set **(B)**.

SUPPLEMENTARY FIGURE S2 DCA of AutoGluon model for day 1 on test set **(A)** and validation set **(B)**.

SUPPLEMENTARY FIGURE S3 Calibration curve of AutoGluon model for day 2 on test set **(A)** and validation set **(B)**.

SUPPLEMENTARY FIGURE S4 DCA of AutoGluon model for day 2 on test set **(A)** and validation set **(B)**.
