# Supplementary figures and images for: CD3+ T-cell count prediction for anti-thymocyte globulin treatment monitorization in kidney transplant recipients: a machine learning model
Source: Front Med (Lausanne). 2026 Jun 18;13:1869846. doi: 10.3389/fmed.2026.1869846 (PMC13322851; doi:10.3389/fmed.2026.1869846)

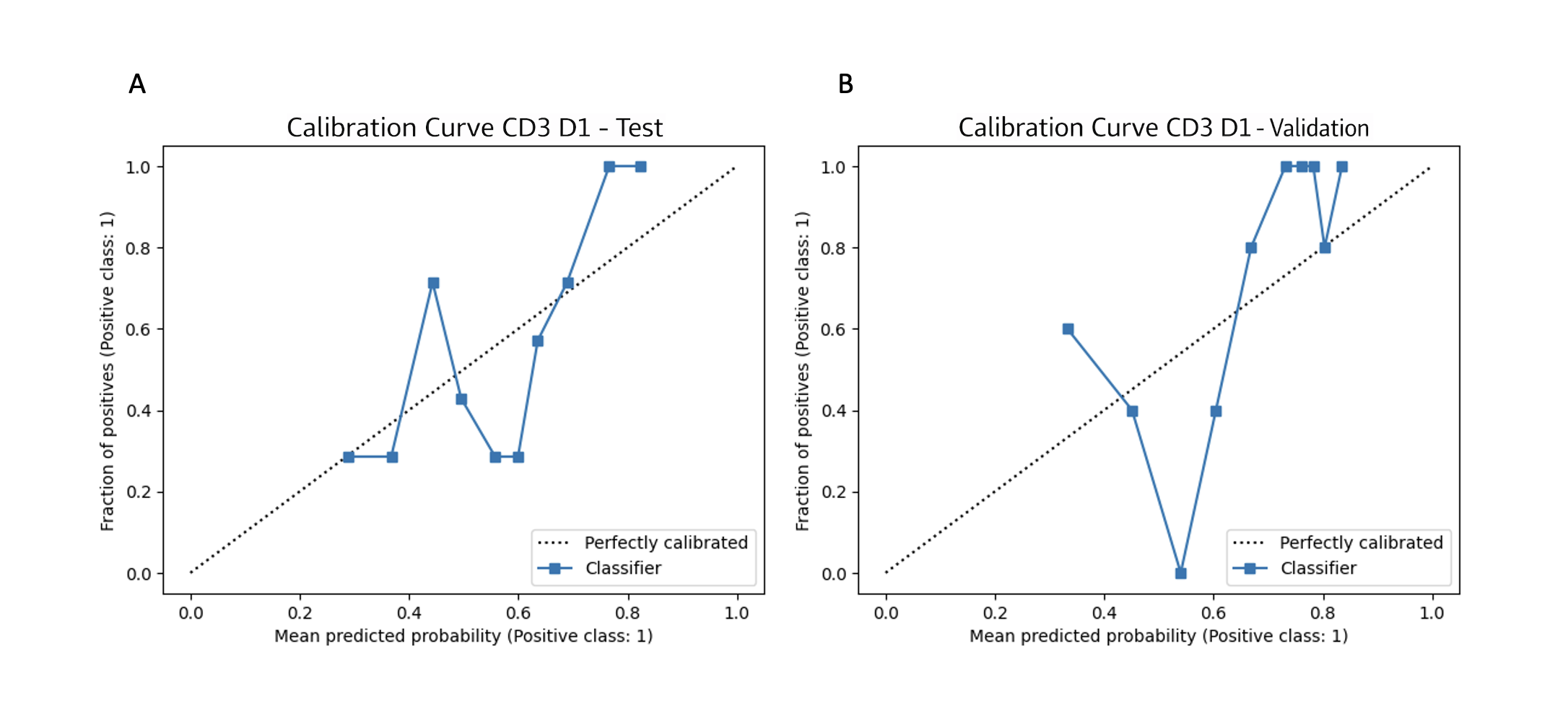

Supplement: SUPPLEMENTARY FIGURE S1 — Calibration curve of AutoGluon model for day 1 on test set (A) and validation set (B). [file Image_1.PNG]

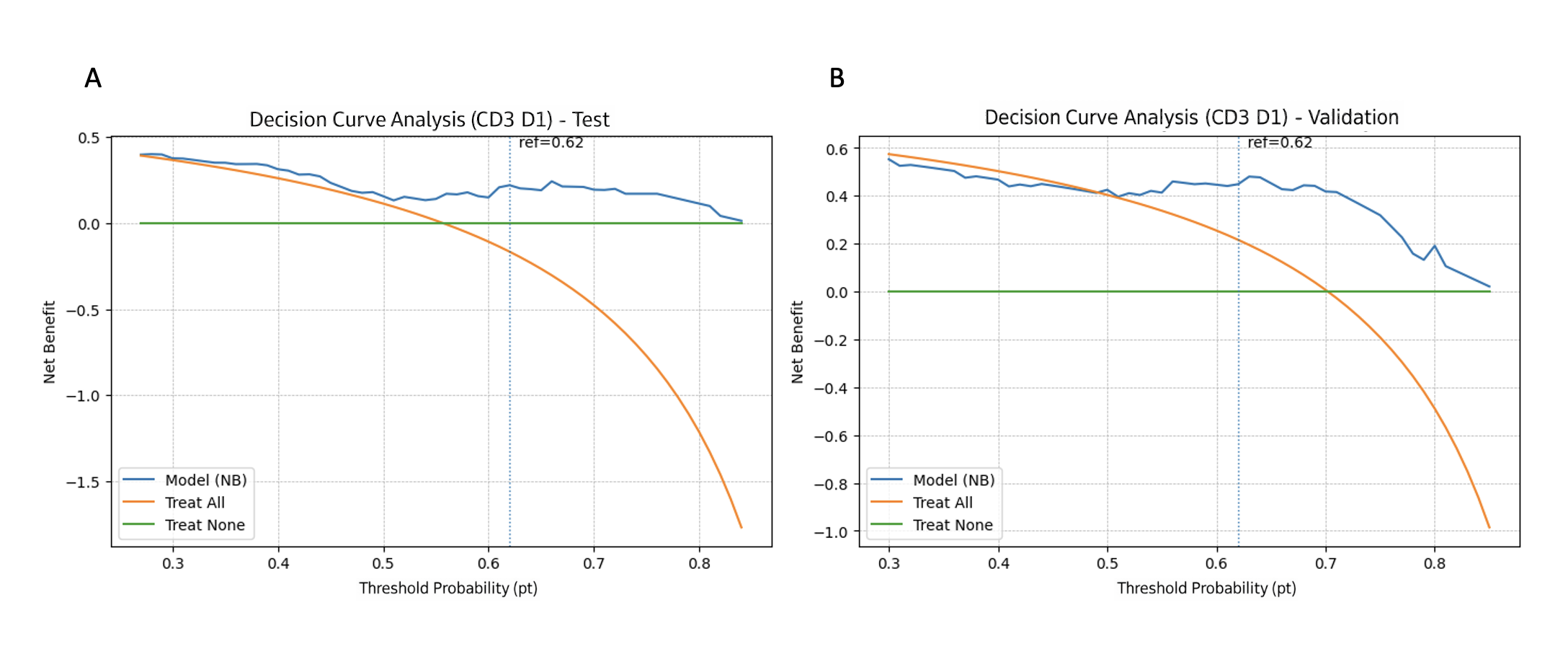

Supplement: SUPPLEMENTARY FIGURE S2 — DCA of AutoGluon model for day 1 on test set (A) and validation set (B). [file Image_2.PNG]

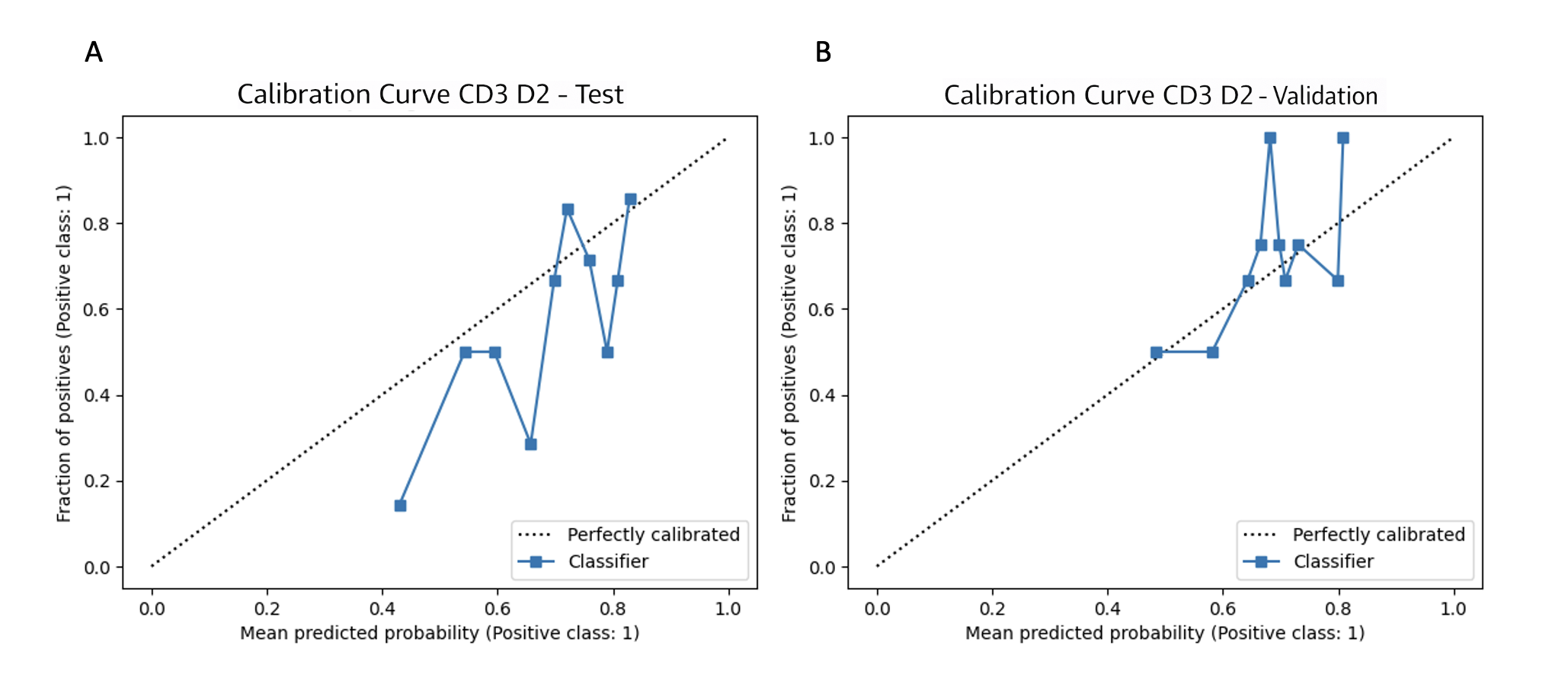

Supplement: SUPPLEMENTARY FIGURE S3 — Calibration curve of AutoGluon model for day 2 on test set (A) and validation set (B). [file Image_3.PNG]

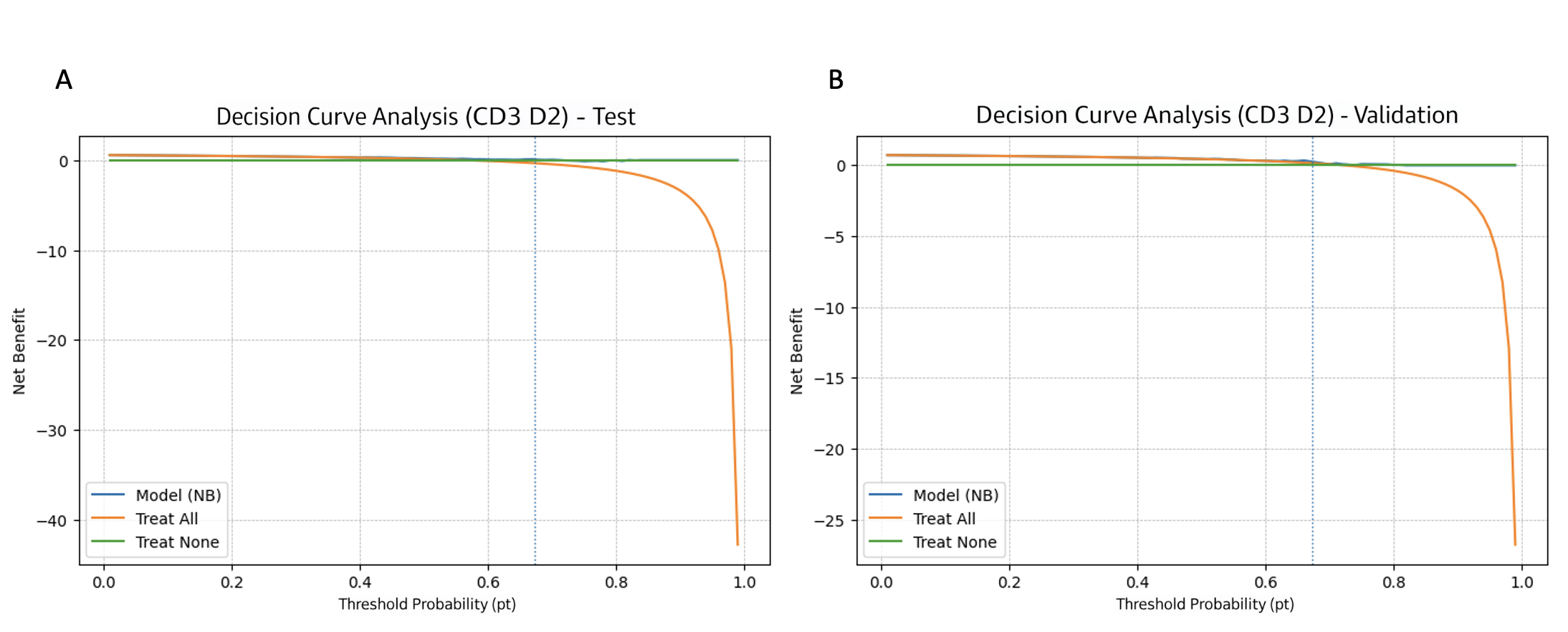

Supplement: SUPPLEMENTARY FIGURE S4 — DCA of AutoGluon model for day 2 on test set (A) and validation set (B). [file Image_4.PNG]
