# Supplementary material for: CD3+ T-cell count prediction for anti-thymocyte globulin treatment monitorization in kidney transplant recipients: a machine learning model
Source: Front Med (Lausanne). 2026 Jun 18;13:1869846. doi: 10.3389/fmed.2026.1869846 (PMC13322851; doi:10.3389/fmed.2026.1869846)
Supplement: Supplementary file 5 [file Table_1.DOCX]

| Comparison of Model Predictive Performance | | | | | | | | |
| --- | --- | --- | --- | --- | --- | --- | --- | --- |
|  |  | ROC-AUC | Sensitivity | Specificity | PPV | NPV | Accuracy | Youden |
| ML Day 1 | Test Set | 0.75 | %58.97 | %87.10 | %85.19 | %62.79 | %71.43 | 0.46 |
|  | Internal Validation Set | 0.80 | %78.79 | %78.57 | %89.66 | %61.11 | %78.72 | 0.57 |
| ML Day 2 | Test Set | 0.71 | %80.56 | %60.71 | %72.50 | %70.83 | %71.88 | 0.41 |
|  | Internal Validation Set | 0.65 | %69.23 | %60.00 | %81.82 | %42.86 | %66.67 | 0.29 |
| LR Day 1 | Test Set | 0.71 | %64.10 | %74.19 | %75.76 | %62.16 | %68.57 | 0.38 |
|  | Internal Validation Set | 0.66 | %81.82 | %57.14 | %81.82 | %57.14 | %74.47 | 0.39 |
| LR Day 2 | Test Set | 0.61 | %75.00 | %46.43 | %64.29 | %59.09 | %62.50 | 0.21 |
|  | Internal Validation Set | 0.48 | %69.23 | %30.00 | %72.00 | %27.27 | %58.33 | (–)0.01 |
