# Supplementary material for: CD3+ T-cell count prediction for anti-thymocyte globulin treatment monitorization in kidney transplant recipients: a machine learning model
Source: Front Med (Lausanne). 2026 Jun 18;13:1869846. doi: 10.3389/fmed.2026.1869846 (PMC13322851; doi:10.3389/fmed.2026.1869846)
Supplement: Supplementary file 6 [file Table_2.DOCX]

| Comparison of Model Predictive Performance with Bootstrap | | | | | | | | |
| --- | --- | --- | --- | --- | --- | --- | --- | --- |
|  |  | ROC-AUC | Sensitivity | Specificity | PPV | NPV | Accuracy | Youden |
| ML Day 1 | Test Set | 0.75 (0.63 – 0.86) | 0.62 (0.47 – 0.76) | 0.70 (0.54 – 0.86) | 0.73 (0.58 – 0.87) | 0.59 (0.44 – 0.74) | 0.66 (0.54 – 0.76) | 0.32 (0.09 – 0.53) |
|  | Internal Validation Set | 0.80 (0.65 – 0.93) | 0.82 (0.68 – 0.94) | 0.71 (0.45 – 0.93) | 0.87 (0.74 – 0.97) | 0.62 (0.36 – 0.85) | 0.79 (0.66 – 0.89) | 0.53 (0.24 – 0.80) |
| ML Day 2 | Test Set | 0.70 (0.57 – 0.83) | 0.80 (0.68 – 0.91) | 0.60 (0.42 – 0.79) | 0.72 (0.57 – 0.85) | 0.70 (0.52 – 0.87) | 0.72 (0.61 – 0.83) | 0.41 (0.19 – 0.63) |
|  | Internal Validation Set | 0.66 (0.41 – 0.87) | 0.70 (0.52 – 0.86) | 0.60 (0.25 – 0.90) | 0.82 (0.65 – 0.96) | 0.43 (0.16 – 0.69) | 0.67 (0.50 – 0.81) | 0.30 (–0.11 – 0.65) |
| LR Day 1 | Test Set | 0.71 (0.58 – 0.82) | 0.64 (0.49 – 0.78) | 0.74 (0.58 – 0.89) | 0.76 (0.61 – 0.90) | 0.62 (0.46 – 0.78) | 0.68 (0.57 – 0.79) | 0.38 (0.15 – 0.58) |
|  | Internal Validation Set | 0.67 (0.49 – 0.84) | 0.82 (0.68 – 0.94) | 0.58 (0.31 – 0.83) | 0.82 (0.69 – 0.949 | 0.57 (0.29 – 0.82) | 0.75 (0.64 – 0.87) | 0.39 (0.10 – 0.69) |
| LR Day 2 | Test Set | 0.61 (0.47 – 0.74) | 0.75 (0.61 – 0.89) | 0.46 (0.28 – 0.66) | 0.64 (0.50 – 0.79) | 0.59 (0.38 – 0.79) | 0.62 (0.52 – 0.75) | 0.21 (–0.04 – 0.46) |
|  | Internal Validation Set | 0.41 (0.15 – 0.67) | 0.73 (0.54 – 0.89) | 0.30 (0.00 – 0.62) | 0.73 (0.54 – 0.89) | 0.30 (0.00 – 0.62) | 0.61 (0.44 – 0.78) | 0.03 (–0.31 – 0.41) |
